# Supplementary material for: Using community-based, participatory qualitative research to identify determinants of routine vaccination drop-out for children under 2 in Lilongwe and Mzimba North Districts, Malawi
Source: BMJ Open. 2024 Feb 1;14(2):e080797. doi: 10.1136/bmjopen-2023-080797 (PMC10836352; doi:10.1136/bmjopen-2023-080797)
Supplement: Supplementary data [file bmjopen-2023-080797supp001.pdf]

Appendix A: Malawi routine vaccination schedule, as of July, 2022

Vaccine	Dose	Recommended age of administration
Tuberculosis	BCG	At birth
Oral Polio	OPV 0	At birth
	OPV 1	6 weeks
	OPV 2	10 weeks
	OPV 3	14 weeks
Inactivated Polio	IPV	14 weeks
Pentavalent: diphtheria, pertussis, tetanus, hepatitis B, haemophilus influenzae type B	DPT-HepB-Hib 1	6 weeks
	DPT-HepB-Hib 2	10 weeks
	DPT-HepB-Hib 3	14 weeks
Pneumococcal conjugate	PCV 1	6 weeks
	PCV 2	10 weeks
	PCV 3	14 weeks
Rotavirus	RV 1	6 weeks
	RV 2	10 weeks
Measles-Rubella	MR 1	9 months
	MR 2	15 months
