# Supplementary figures and images for: Using community-based, participatory qualitative research to identify determinants of routine vaccination drop-out for children under 2 in Lilongwe and Mzimba North Districts, Malawi
Source: BMJ Open. 2024 Feb 1;14(2):e080797. doi: 10.1136/bmjopen-2023-080797 (PMC10836352; doi:10.1136/bmjopen-2023-080797)

Appendix B: Adapted Behavioral and Social Drivers (BeSD) of Vaccination Model

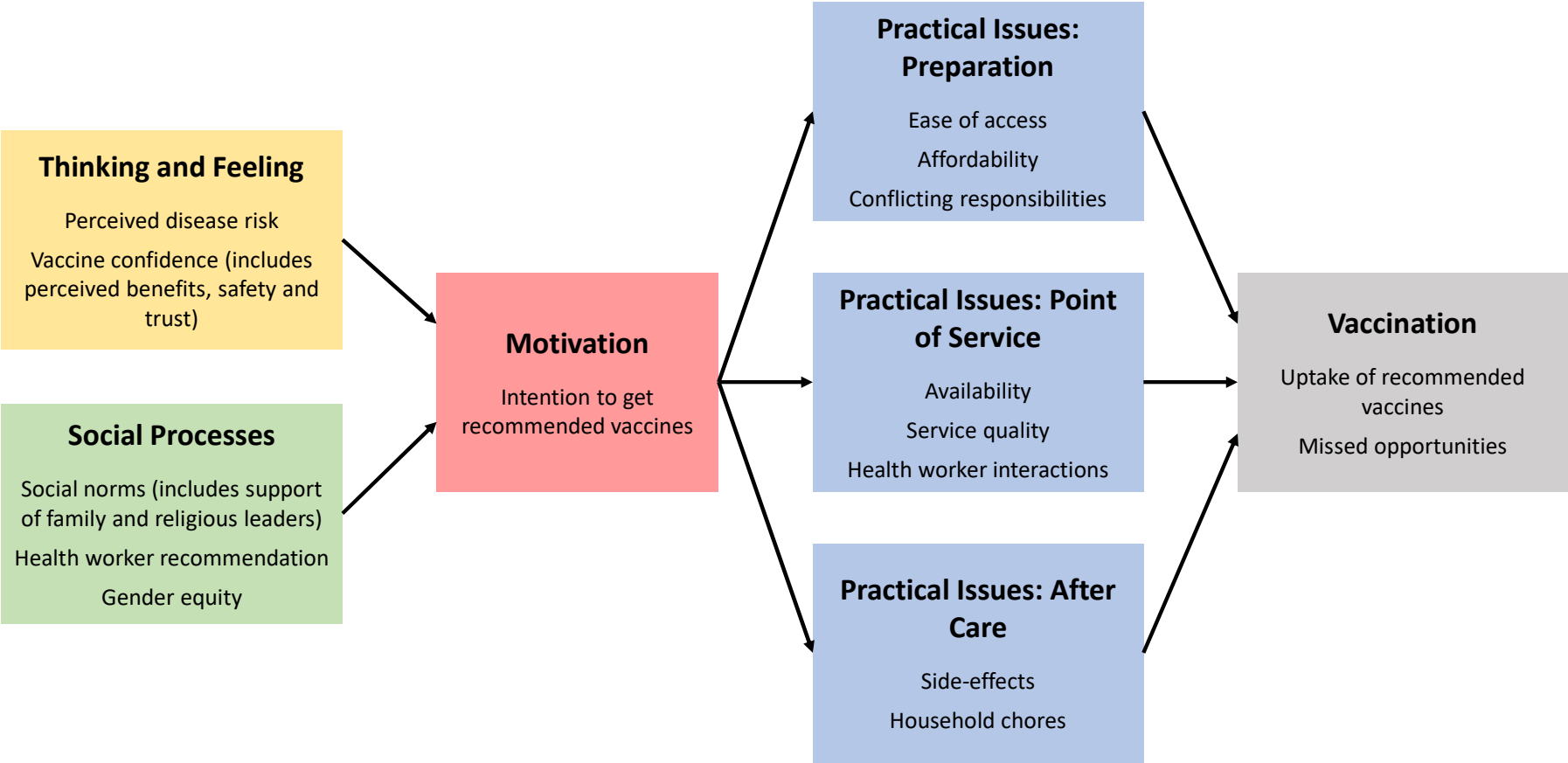

Supplement: Supplementary data [file bmjopen-2023-080797supp002.pdf]
