# Supplementary material for: Using community-based, participatory qualitative research to identify determinants of routine vaccination drop-out for children under 2 in Lilongwe and Mzimba North Districts, Malawi
Source: BMJ Open. 2024 Feb 1;14(2):e080797. doi: 10.1136/bmjopen-2023-080797 (PMC10836352; doi:10.1136/bmjopen-2023-080797)
Supplement: Supplementary data [file bmjopen-2023-080797supp003.pdf]

Appendix C: Photo Elicitation In-Depth Interview Guide

Photo Elicitation In-Depth Interview Guide

Introduction:

Introduction: My name is _____________, and I work as a Caregiver Researcher with VillageReach and the Ministry of Health. VillageReach is a non-profit organization that works with governments and other partners to expand healthcare access to difficult-to-reach communities. I would like to thank you very much for agreeing to participate in this research activity to share your experiences vaccinating [child's name]. Do you have any questions?

Verify participant information:
Now, before we get started can I verify your information?
[if yes]

- ☐ Child's name matches the name and information on the recruitment sheet
- ☐ Child's vaccination card matches the vaccination records on the recruitment sheet
    - *If the card does not match the health facility records, use the card as the "gold standard" and classify the child's vaccination status based on the card*
- ☐ [If part of the fully vaccinated group] Receive verbal confirmation that the child received all vaccinations before age two
    - *If no, participant is not eligible for the study*
- ☐ [If part of the partially vaccinated group] receive verbal confirmation that the child received at least one but not all vaccinations before age two.
    - *If no, participant is not eligible for the study*
- ☐ Confirm that the participant is the primary caregiver by asking, "Are you the one who takes the primary responsibility of bringing the child to access health services."
    - *If no, ask them to direct you to the primary caregiver for recruitment in the study*

If meet eligibility criteria, continue to consent.

Identifying scalable community-identified vaccination solutions: A multi-site community participatory based study to identify drivers and scalable solutions of vaccination access and uptake

Semi-Structured Discussion Guide for Photo Elicitation In-depth Interview

Instructions to Caregiver Researchers

- *If you are continuing with the in-depth interview it means the participant has agreed to participate in the study. Please make sure you have their signed consent to participate and record the interview. Give them their copy of the consent and file your copy.*
- *Ask them if they have any questions or if they would like you to read the informed consent again.*
- *Please do not answer your phone while you are conducting the interview.*

Introduction to In-depth Interview

- The interview will last about 1.5 hours.
- Please know that what you say to us is confidential. We know how important this is to you. We also ask that the other participants keep as confidential what we discuss here.
- To better understand vaccination in your community, it is really important that you share your true beliefs and attitudes towards vaccination, as well as your personal experiences in vaccination of your children.

Before we begin, let me collect a few pieces of information from you.

- *Ask for the following demographic information and register it in the "data collection tracking" sheet:*
  - Age
  - Gender
  - Education level
  - Urban/rural residence
  - Relationship with child 25-34 months (e.g. father or mother, grandfather/grandmother, aunt/uncle, etc.)
  - Number of children under 10 years of age in the household

Do you have any other questions before we get started?

Discussion Guide for Photo Elicitation: The first part of this study is to understand your experience in vaccinating [child's name], by showing you photos of things that relate to your experience of vaccinating [child's name]. Do you have any questions?

Photo Brainstorming and research question: Now let's discuss together to think of a few ideas for photos that relate to your experience of vaccinating [participant's child's name] when he/she was a new-born to when he/she was two years old.

Identifying scalable community-identified vaccination solutions: A multi-site community participatory based study to identify drivers and scalable solutions of vaccination access and uptake

- Do you have a copy of your child's vaccination card that we look at as you tell me about your experience vaccinating [child's name]? *If yes, ask them to bring the vaccination card. If no, use the vaccination register data as a reference.*

[Turn on recorder and start photo brainstorming discussion]

- Can you describe your personal experience with the process of vaccinating [child's name] for each vaccination? [Go through each vaccination on the card and/or on the vaccination calendar]

  Probes:

  - *Did you have to make any special preparations or plans before you could bring [child's name] for vaccinations?*
  - *If yes, how did these special plans impact your everyday life?*
  - *Were there any major events that happened in your family during those first two years of your child's life? (E.g. deaths, illnesses, employment loss or gain, family members coming or leaving)*
  - *If yes, how did these events fit into your child's vaccination schedule?*
  - *Can you describe what was your experience like on the days you brought your child for vaccination?*
  - *[If they got vaccinations more than once] Were your child's vaccination experiences similar or different from each other? How so?*

- *[When you get to a point where a vaccination was missed, ask "what happened here?"]*

Now that we've mapped out some of your personal experiences with vaccinating [child's name], can you think of any pictures that might represent different aspects of that experience?

Probes:

- *If child is partially vaccinated ask – what things could represent in a photo reasons why [child's name] didn't get all of their vaccines by age two?*
- *If fully vaccinated, ask- what things could represent in a photo why [child's name] is fully vaccinated?*
- *What things could represent in a photo what was easy about trying to immunize [child's name]?*
- *What things could represent in a photo what the experience of accessing vaccination services for [child's name] felt like?*
- *What things could represent in a photo the things/factors you considered when vaccinating [child's name]?*
- *What things could represent in a photo what was hard about trying to immunize [child's name]?*

[Pause the recorder to review the photos]

Identifying scalable community-identified vaccination solutions: A multi-site community participatory based study to identify drivers and scalable solutions of vaccination access and uptake

Review of Photos:

- ☐ Show them the photos you have (show them the photos one at a time and start by asking: what they think the photo is depicting related to the vaccination journey).
- ☐ Lay out all of the photos and ask them to pick the ones they resonate most/ represent their experience of vaccinating [child's name].
- ☐ Record the ID numbers of the 5 photos that the caregiver selected in the "data collection tracking sheet".
- ☐ Begin the interview using the interview guide

[Start the recorder again for the in-depth interviews]

Semi-Structured Discussion Guide for Photo Elicitation In-depth Interview

Instructions: To elicit rich descriptions from participants, when asking follow up questions or encouraging them to talk more or give you more examples/details use the following probes:

- *What do you mean by ____ ?*
- *Tell me more about ____.*
- *Can you give me an example of ____ ?*
- *Can you tell me about a time when ____ ?*
- *Can you tell me about the last time ____ ?*
- *Is there anything that made ____ easier?*
- *Is there anything that made ____ harder?*
- *(Can you tell me) Who ____?*
- *(Can you tell me) When ____ ?*
- *(Can you tell me) Where ____ ?*

Probes are completed using <u>only</u> verbatim participant words or phrases.

Interview Guide:

Once photos are selected, ***ask for each photo:***

- What do we see in this photo?
- What does this represent to you?
- How does this photo represent your vaccination journey?

Now, based on our activity with the photos, we have learned more about your experience, thank you. Now we have some more questions to better understand your experience vaccinating your child vaccination and the things that influenced [child's name] vaccination status.

Identifying scalable community-identified vaccination solutions: A multi-site community participatory based study to identify drivers and scalable solutions of vaccination access and uptake

[Instruct to use past interview/photos as prompts in the interview]

Knowledge, Beliefs, and Perceptions about Vaccination

1. To start, can you tell me about how you feel about vaccination?
    a. How do other people in your family talk about vaccination?
        i. How does this relate to how you feel about vaccinating [child's name]?
    b. How do other people in your community talk about vaccination?
        i. How does this relate to how you feel about vaccinating [child's name]?
    c. Have your feelings about vaccination changed at all with COVID-19?
        i. If yes, why and how?
        ii. If no, why not?

2. What kind of information, if at all, did you receive about vaccinating your child or vaccinations in general?
    a. When did you receive this information?
    b. Where did this information come from?
        i. Health workers (at the facility vs. outreach in the community?)
        ii. HSAs? (at the facility vs. outreach in the community?)
        iii. Social media (Facebook, WhatsApp, other)?
        iv. Neighbours?
        v. Relatives?
        vi. Radio?
        vii. Community, religious, school leaders?
        viii. Vaccination campaigns?
    c. How often, if at all, did you receive information?
    d. To what extent did you trust this information?
    e. Did you get information about which vaccinations your child needed and when they were needed?
        i. How confident did you feel in your knowledge about when to bring [child's name] for vaccines and how many vaccines [child's name] needed?
    f. Did you get information about what to do if your child has missed a vaccination?
        i. If so, how confident did you feel in your knowledge about what to do if your child has missed a vaccine?
    g. Did you get information about the side effects your child might experience after vaccinations?
    h. Did COVID-19 change the type of information you receive about vaccinations or how/where you receive that information? How?

Identifying scalable community-identified vaccination solutions: A multi-site community participatory based study to identify drivers and scalable solutions of vaccination access and uptake

Decision-Making Processes and Motivation to Vaccinate

3. What factors influenced your decisions about vaccinating [child's name]?
    a. Who was involved with making those decisions? Was there anyone who helped you make that decision?
        i. How so?
        ii. Who is the final decision maker about whether [child's name] should be vaccinated?
    b. When you began [child's name's] vaccinations, did you have any plans or goals related to [child's name's] vaccinations?
        i. If yes, what were your goals?
            1. *Probe on:*
                a. *Completing all vaccinations*
                b. *Completing certain vaccinations*
                c. *Staying on schedule*
        ii. If no, why not?
        iii. How important did you feel getting these vaccinations were, if at all, for [*child's name*]? Why?
        iv. Was there anyone who influenced your feelings about whether or not to fully vaccinate your child? Who? How did they influence you?
    c. Did you have questions or concerns about the recommendations for vaccinating [*child's name*]?
        i. Why or why not?
    d. Have any of these factors changed since COVID-19?
        i. If so, how?

Vaccination Experience

4. I'd like to hear more about [child's name's] vaccination experiences.
    a. Where did [child's name] get their vaccinations?
        i. Did they get them all there?
        ii. What did you think about getting the vaccinations there?
    b. Can you describe your interactions with the health staff who administered [child's name's] vaccines?
        i. Who administered [child's name's] vaccines?
        ii. How do you feel about that?

Identifying scalable community-identified vaccination solutions: A multi-site community participatory based study to identify drivers and scalable solutions of vaccination access and uptake

iii. Did you trust that [HSA/health worker/ nurse/doctor] who gave the vaccinations?

1. Why or why not?

iv. Were you able to speak to that [HAS/health worker/ nurse / doctor] when you had questions about [child's name's] vaccinations?

1. How did they respond to your questions?

c. How satisfied were you with the experience(s) of vaccinating [child's name]?

i. What lead to you feeling this satisfied/dissatisfied?

d. Can you describe your experiences after [child's name] received the vaccine?

i. What was your experience after the vaccination?

ii. What was your child's experience after the vaccination?

Vaccination Dropout (ask these questions only for those who have a child who dropped out)

5. *[for those who have a child who dropped out]* Can you describe why [child's name] didn't receive all their vaccinations by age two?

a. What factors do you think contributed to that?

i. Probe on:

1. What kind of information did you receive about your child's vaccination schedule, if at all?

2. Did you know what your child's vaccination schedule was?

3. Did COVID-19 effect your ability to continue with your child's vaccinations? How?

b. Did you want [child's name] to complete their vaccinations?

i. Why or why not?

c. What was hard about your experiences getting [child's name] vaccinated?

i. Can you provide an example?

ii. Did COVID-19 make your child's vaccination experiences harder in any ways? How

d. Was there anything easy about your experiences getting [child's name] vaccinated?

i. Can you provide an example?

ii. Did COVID-19 make your child's vaccination experiences easier in any ways? How?

e. Can you describe your experiences after [child's name] did not complete their vaccinations?

i. How did your family and community react to [child's name] not completing their vaccinations?

ii. How did you feel about [child's name] not completing their vaccinations? Why?

Identifying scalable community-identified vaccination solutions: A multi-site community participatory based study to identify drivers and scalable solutions of vaccination access and uptake

Vaccination Completion (ask these questions only for those who have a child who completed vaccination)

6. [for those who have a fully vaccinated child] What factors do you think contributed to being able to fully immunize [child's name] by the age of two?
    a. What was hard about your experiences getting [child's name] vaccinated?
        i. Can you provide an example?
        ii. Did COVID-19 make your child's vaccination experiences harder in any ways? How?
    b. What was easy about your experiences getting [child's name] vaccinated?
        i. Can you provide an example?
        ii. Did COVID-19 make your child's vaccination experiences easier in any ways? How?
    c. Can you describe your experiences after [child's name] completed their vaccinations?
        i. How did your family and community react to [child's name] completing their vaccinations?
        ii. How did you feel about [child's name] completing their vaccinations? Why?

Reflection on Vaccination Experience (for all caregivers)

7. How do you wish the experience of getting all the recommended vaccinations for [child's name] could be different, if at all?
    a. What would you change? Would this make the vaccination experience easier? How?
    b. What would you keep the same? Why?
    c. If you could go through your child's vaccination process again, is there anything you would do differently?

Conclusion: Are there any final thoughts you wish to share about your experiences with vaccinating your 2-3 year old child?

After we finish conducting all of the interviews, we plan to have a workshop to bring caregivers, health workers, community leaders, and other community members together to discuss solutions to make the vaccination experience easier. Would you be willing to participate in this workshop?

Yes:____ No:____

[If yes, ask for caregiver's preferred contact details to follow-up about workshop participation]

Contact information for caregiver:

We would like to thank you for taking the time to discuss this important topic with us.
