# Supplementary material for: Using community-based, participatory qualitative research to identify determinants of routine vaccination drop-out for children under 2 in Lilongwe and Mzimba North Districts, Malawi
Source: BMJ Open. 2024 Feb 1;14(2):e080797. doi: 10.1136/bmjopen-2023-080797 (PMC10836352; doi:10.1136/bmjopen-2023-080797)
Supplement: Supplementary data [file bmjopen-2023-080797supp004.pdf]

Appendix D: Description of Photo Set Used for Photo-Elicitation Data Collection

**Category / Theme**	**Descriptions of Individual Photos**
Path / walking to facility (5 photos)	Caregiver and child sitting under a tree along the path to/from the facility
	Caregiver walking on a trail to facility with one child on back and another child walking beside her
	Group of three caregivers carrying children and walking to/from the facility together
	Caregiver walking on dirt road with child on back
	Caregiver walking across a river while carrying two children
Child needing rest or care after vaccination (3 photos)	Caregiver sitting on a mat next to child
	Child laying down on a mat
	Caregiver and child lying down next to each other on a mat
Transport to facility (3 photos)	Caregiver next to male relative / friend with a bicycle
	Male sitting on motorbike with two children on the back
	Caregiver standing in front of a motorbike
COVID-19 (2 photos)	Caregiver washing hands at the entrance to the facility
	Caregiver wearing face mask
Healthy child (1 photo)	Child smiling
Child eating after vaccination (2 photos)	Caregiver and child sitting together on mat with plates of food
	Child sitting on mat eating food
	Mother and father sitting outside the facility with child

Family or husband support (5 photos)	Father reviewing child's vaccination card at home
	Grandmother sitting on mat and caring for child
	Multiple relatives sitting and caring for child
	Relative sitting with child while caregiver does laundry
Bathing child (1 photo)	Caregiver bathing child in plastic tub
Household chores (5 photos)	Caregiver cooking
	Water jugs next to river
	Caregiver cleaning dishes in river with child sitting next to her
	Caregiver doing laundry
	Caregiver filling water bucket from tap with child on her back
Health worker / health facility (2 photos)	Health personnel injecting vaccine while caregiver holds child
	Caregiver walking into health facility with child on back
