# Supplementary material for: Using community-based, participatory qualitative research to identify determinants of routine vaccination drop-out for children under 2 in Lilongwe and Mzimba North Districts, Malawi
Source: BMJ Open. 2024 Feb 1;14(2):e080797. doi: 10.1136/bmjopen-2023-080797 (PMC10836352; doi:10.1136/bmjopen-2023-080797)
Supplement: Supplementary data [file bmjopen-2023-080797supp005.pdf]

Appendix E: Immunization Clinic Observation Guide

Immunization Clinic Observation Guide

Instructions:

- The purpose of this guide is to gain an insight into how an under-2 immunization session is conducted at the health facility or an outreach clinic. Before you begin the session, please make sure you have a copy of this guide and a notepad and pen to write down your observations.
- Prior to the observation, you (caregiver researcher) will ask for a verbal consent from the health facility in-charge and the Health Care Worker (HCW) conducting the immunization session.
- Observe the whole immunization clinic session from start to finish
- Record all your observations in your study notes, and after the session, do the following
    - Type all notes you made during the observation
    - Rename any pictures you took with: District_Date_health facility name_Observation#
    - Upload in the dropbox folder and inform Joan
    - Delete the picture in your phone/computer

Clinic type (circle which applies): Outreach activity Health facility

Health Care worker: [LIST types of workers)

Observe and note the following key things and anything that seems to standout/ unusual

1. The general environment/atmosphere of the vaccination clinic
    a. Rough estimate of the number of children under 2
    b. Number of children vaccinated during the session (ask the HWC if this was more, less or the same than normal- did today seem like a busy or a slow day?)
    c. Time the vaccination session starts and ends
    d. Number of HCW vaccinating the children
    e. Clinic set up
        i. How private is the area where the child is getting the vaccine?
        ii. Is there any waiting place? What does it look like? How crowded is it?
        iii. How is the environment?
        iv. Where is the waiting area? Is it protected from rain / sun?

2. The process:
    a. What happens before they start vaccinating the children?
        i. General health messaging/ vaccination awareness sessions (Health talks)?
        ii. If awareness (health talk) is conducted, what type of messages are they disseminating?
        iii. Have most of the caregivers arrived before the start of the awareness session, or are there caregivers who arrive later and miss the session?
    b. What happens during the vaccination session?
        i. Observe if they are available through the clinic session or if they are taking any breaks, if so, how long, to do what?
        ii. Is the HCW busy with their phone during the session? Texting, answering calls, etc
        iii. How is the HCW interaction with the caregivers?

iv. Are they chatting/not? Is the HCW giving personalized health messaging to the caregiver for example, focusing on side effects or next vaccination schedule/date?
v. How is the HCW attitude to the caregivers for example, attentive, giving attention to the caregiver, rude, etc?
vi. How much time does the HCW spend with each caregiver? Are they taking enough time to explain the vaccination process?
vii. Does the HCW ask if the caregiver has any questions? Does the caregiver ask the HCW any questions? If so, how does the HCW respond?
viii. Is there a standard process or are there any inconsistences from one child to the next? E.g., recording in immunization cards, recording in the register, following a checklist or an SOP?

c. What other activities is the HCW involved in besides vaccinating the children?
d. Any coordination you are seeing with other HCW? For example, referral to the curative clinic for other services and/or referral from the curative to immunization clinic?
e. Is there anything you think is delaying/hurrying the service or making it go smooth?
f. Are any caregivers sent back home without immunizing their children? If so, why – vaccine stock-out, late for vaccination clinic (Under 5 clinic), not due for next vaccination, missing immunization card, unclean child? If a caregiver comes without the immunization card, what happens next?
g. If it's an outreach clinic, is the community involved? If so, how? Who from the community is involved? How so?
h. Anything else you are able to observe that is not addressed yet?
