# Supplementary material for: Using community-based, participatory qualitative research to identify determinants of routine vaccination drop-out for children under 2 in Lilongwe and Mzimba North Districts, Malawi
Source: BMJ Open. 2024 Feb 1;14(2):e080797. doi: 10.1136/bmjopen-2023-080797 (PMC10836352; doi:10.1136/bmjopen-2023-080797)
Supplement: Supplementary data [file bmjopen-2023-080797supp006.pdf]

Appendix F: Semi-Structured Discussion Guide for Healthcare Worker Interviews

Semi-Structured Discussion Guide for Healthcare Worker Interviews

Instructions for Caregiver Researchers: The purpose of these interviews is to learn more about the role that HSAs play in the childhood immunization process and to hear their perspectives on some of the causes of immunization dropouts. This is also an opportunity to probe for more information on anything notable that arose from the Telegram exchanges you had with that HSA or during the observations at the facility. This is just a rough guide of questions to ask the healthcare workers, and additional questions or topics can be discussed based on specific observations that you made from the messages. Before you go into the interview, please review the Telegram exchanges you had with that HSA and make note of any interesting points or observations they brought up so that you can discuss them further during this interview. Also make note of anything notable that you observed during the observation session and follow-up on it during the interview.

Introduction:

Thank you for taking the time to talk in more detail about your experiences and perspectives on under-2 immunizations. During this interview, I want to learn more about your role delivering vaccinations and interacting with caregivers and the factors that you think could relate to immunization dropout. We'll also talk a bit about some of the message exchanges we had on Telegram and on some of the observations from this morning so that I can learn about those in more detail. As a reminder, everything you say will remain confidential and anonymous. Before we begin, do you have any questions?

Interview Questions

1) Now, I am going to walk through some of the observations you mentioned in your messages over the last few weeks as well as some of the observations that I made at the facility today, and I would like to get your perspective on:
    a) Were these typical [experience, process, observation]?
        i) If so, how? If not, how did they vary from normal?
    b) Is there anything else you would like to share or explain about the [experience, process, observation] that may not have been captured in the messages?
2) How do you see your role in immunizations?
    a) What are your personal beliefs around vaccinations?

   b) How do you feel about that role?

   c) Do you feel supported in your role? If not, why? If yes, how so?

   d) How do you generally feel about the tasks associated with immunizations?

3) How do you feel about the overall system for immunizations?

   a) How do you feel about how immunization records are kept and reported?

   b) What about management of vaccines?

4) Apart from administering vaccines, what other roles or duties do you perform here at the facility/outreach?

   a) To what extent do the U2 vaccination tasks or role take up your workload?

5) Can you tell me about the supervision, mentorship, and training you receive, if at all?

   a) What supervision / mentorship support do you receive?

      i) Who supervises or mentors you?

      ii) How often do you receive supervision or mentorship?

      iii) To what extent do you feel you are receiving the supervision and mentorship you need to do your job well?

   b) What kind of immunization trainings have you received?

      i) What topics are covered in those trainings?

      ii) How often do you participate in trainings related to immunization?

      iii) To what extent do you feel that you are receiving the training you need to do your job well?

6) Can you tell me more about what motivates you in your job?

   a) Do you receive any incentives relating to vaccination?

   b) [If yes]

      i) What are those incentives?

ii) How do those incentives impact your role in the immunization process?

iii) Do those incentives differ in public vs private facilities?

7) Now, I would like to get a better sense of your work around immunizing children under the age of two. Can you walk through a typical day of conducting immunizations for children under the age of two? [Probe on what activities they do to prepare for immunizations, conduct immunizations, document immunizations, educate around immunization]

For each step/activity?

a) Why you take that particular step

b) Who is typically involved in that step

c) What you feel when going through that step

d) What factors make that step easier/harder

e) Are there any cases where you would not administer a vaccination to a child? Why or why not?

8) Can you describe the conversations you usually have with caregivers while administering vaccines?

a) When do these conversations typically occur?

b) What about how you see your role in providing advice to caregivers to vaccinate their children under the age of two?

c) Can you provide an example of a typical conversation?

9) Are there any policies that you follow related to U2 immunizations either for your particular health facility, district or national policy?

a) Have there been any new policies or procedures in the past year related to under-two immunization? If so what?

b) What do you think the impact of [X] policy is (probe on each policy)

c) What happens if a caregiver arrives to the facility late?

i) Is this a formal practice or informal practice?

ii) Is this something that most HSAs do at your facility?

iii) To what extent do you think this impacts their vaccination experience and/or vaccination status?

d) What happens if a caregiver arrives without a vaccination card?

i) Is this a formal practice or informal practice?

ii) Is this something that most HSAs do at your facility?

iii) How often do caregivers arrive at the facility without a vaccination card?

iv) To what extent do you think this impacts their vaccination experience and/or vaccination status?

e) What happens if a child arrives for treatment of an illness and that child is found to be missing vaccines?

i) To what extent do you think this impacts their vaccination experience and/or vaccination status?

10) What, if at all, is challenging about administering under two vaccinations?

a) Did you encounter any of those challenges over the past month? If so, which ones? How did that make your job more challenging?

b) Can you provide an example?

11) What factors make it easier for you to administer vaccinations?

a) Did you encounter any of those factors over the past month? If so, which ones? How did that make your job easier?

b) Can you provide an example?

12) What are your perspectives on immunization sessions conducted at static versus outreach facilities?

a) What do you feel are the main similarities and differences between immunization sessions at these different locations?

13) What are your perspectives on immunization sessions conducted at public versus private facilities?

a) What do you feel are the main similarities and differences between immunization sessions at these different types of facilities?

b) Have you ever worked at a private facility delivering immunizations? [*If yes*,] What is/was that experience like?

c) What kind of caregivers typically go to private facilities over public facilities? Why?

14) Are there other, non-typical ways that people can access immunizations?

a) Can you tell me about those?

b) Who accesses immunizations through those services? Why?

c) What are the immunization experiences like at those services?

15) Can you tell me about how you think vaccination is perceived in the communities that your facility serves?

a) Are there any community beliefs you are aware of related to vaccinations? What are those?

i) Are they general beliefs or specific to certain groups/individuals in the community? If so, can you describe the characteristics of those groups/individuals?

b) Do you know if there is any stigma in the community around vaccination or non-vaccination?

i) If yes, why do you think this is?

ii) What do you think the impact of this is?

c) Can you tell me about any specific communities or sub-sets of people that you think are unlikely to get vaccinated due to community beliefs or stigmas?

i) E.g. certain religious communities, socioeconomic groups, cultural groups, etc.

16) Can you tell me about any specific communities or sub-sets of people that might face unique challenges or barriers to vaccination?

a) What communities are those?

i) E.g. certain religious communities, malnourished children, socioeconomic groups, cultural groups, etc.

b) What kinds of barriers or challenges do they face related to vaccination?

17) Can you tell me more about how transient or tenant communities access vaccination services? By transient, I mean those individuals that may move seasonally for work, migrant populations or mobile populations?

    a) Do you know how those communities perceive vaccination?

    b) Where do these communities usually go to receive vaccinations?

    c) In what ways do you think the vaccination experiences of transient communities are different from the experiences of non-transient communities?

18) What do you view as the primary drivers of under-two immunization drop-outs?

    a) Can you provide an example?

    b) What recommendations do you have for how the vaccination process can be improved to reduce dropouts?
